# A Dynamic Hybrid Weighting Framework for Teaching Effectiveness Evaluation in Multi-Criteria Decision-Making: Integrating Interval-Valued Intuitionistic Fuzzy AHP and Entropy Triggering

**DOI:** 10.3390/e28020241

**Published:** 2026-02-19

**Authors:** Chengling Lu, Yanxue Zhang

**Affiliations:** School of Electrical and Photoelectric Engineering, West Anhui University, Lu’an 237012, China

**Keywords:** entropy-based weighting, interval-valued intuitionistic fuzzy AHP, teaching effectiveness evaluation, multi-criteria decision-making, fuzzy comprehensive evaluation, uncertainty modeling

## Abstract

Multi-criteria decision-making (MCDM) problems in complex evaluation systems are often characterized by high uncertainty in expert judgments and dynamic variations in indicator importance. Traditional analytic hierarchy process (AHP) and entropy-based weighting methods typically suffer from two inherent limitations: the inability to explicitly quantify expert hesitation and the rigidity of static weight assignment under evolving data distributions. To address these challenges, this paper proposes a dynamic hybrid weighting framework that integrates an interval-valued intuitionistic fuzzy analytic hierarchy process (IVIF-AHP) with an entropy-triggered correction mechanism. First, interval-valued intuitionistic fuzzy numbers are employed to simultaneously model membership, non-membership, and hesitation degrees in pairwise comparisons, enabling a more comprehensive representation of expert uncertainty. Second, an entropy-triggered dynamic fusion strategy is developed by jointly incorporating information entropy and coefficient of variation, allowing adaptive adjustment between subjective expert weights and objective data-driven weights. This mechanism effectively enhances sensitivity to high-dispersion criteria while preserving expert knowledge in low-variability indicators. The proposed framework is formulated in a hierarchical fuzzy decision structure and implemented through a fuzzy comprehensive evaluation process. Its feasibility and robustness are validated through a concrete case study on teaching effectiveness evaluation for a university engineering course, leveraging multi-source data. Comparative analysis demonstrates that the proposed approach effectively mitigates the weight rigidity and evaluation inflation observed in conventional methods. Furthermore, it improves diagnostic resolution and decision stability across different evaluation periods. The results indicate that the proposed entropy-triggered IVIF-AHP framework provides a mathematically sound and practically applicable solution for dynamic MCDM problems under uncertainty, with strong potential for extension to other complex evaluation and decision-support systems.

## 1. Introduction

Multi-criteria decision-making (MCDM) problems are widely encountered in complex evaluation systems involving heterogeneous indicators, multiple stakeholders, and uncertain information sources. Typical application domains include performance assessment, risk evaluation, system diagnosis, and quality analysis, where decision outcomes are strongly influenced by both expert judgments and data-driven evidence. In such contexts, two fundamental challenges persist: the inherent uncertainty and hesitation in human expert assessments, and the dynamic variation of indicator importance caused by evolving data distributions and system conditions [1,2,3,4].

The analytic hierarchy process (AHP) and its extensions have been extensively applied to derive criterion weights in MCDM problems due to their clear hierarchical structure and interpretability [5]. However, conventional AHP relies on precise pairwise comparisons and fixed numerical scales, which are often insufficient to capture the ambiguity, hesitation, and partial confidence inherent in expert judgments [6,7,8]. To address this limitation, intuitionistic fuzzy sets and interval-valued intuitionistic fuzzy numbers (IVIFNs) have been introduced to enhance the expressive capability of AHP by simultaneously modeling membership, non-membership, and hesitation degrees. Despite these advances, most existing IVIF-AHP-based approaches remain fundamentally static, assuming that expert-derived weights are invariant once determined.

In parallel, entropy-based weighting methods have been proposed as objective alternatives that extract indicator importance from data dispersion characteristics [9]. Information entropy reflects the uncertainty or disorder of evaluation data, while extensions incorporating statistical measures such as the coefficient of variation further enhance sensitivity to heterogeneous distributions [10]. Nevertheless, entropy-based methods are typically applied in a fixed or linear manner and often neglect expert knowledge, which may result in unstable or counterintuitive weights in low-variability indicators or small-sample scenarios [11].

The coexistence of subjective expert uncertainty and objective data variability highlights a structural deficiency in many existing MCDM frameworks: the lack of an adaptive mechanism that dynamically regulates the contribution of expert-driven and data-driven weights according to indicator-specific information characteristics [9,12,13]. In complex evaluation systems, indicators with high dispersion should exert greater influence on decision outcomes, whereas indicators with low variability but high expert consensus should preserve the dominance of expert knowledge. Static weighting schemes, whether subjective or objective, are inherently incapable of accommodating such heterogeneous behaviors [14,15].

To overcome these limitations, this paper proposes a dynamic hybrid weighting framework that integrates an interval-valued intuitionistic fuzzy analytic hierarchy process (IVIF-AHP) with an entropy-triggered correction mechanism. IVIF-AHP is employed to construct initial subjective weights while explicitly quantifying expert hesitation and disagreement. Subsequently, an entropy-trigger strategy combining information entropy and coefficient of variation is developed to adaptively adjust the fusion ratio between subjective and objective weights at the indicator level. The proposed framework is embedded within a hierarchical fuzzy comprehensive evaluation structure to support systematic aggregation and diagnostic analysis in complex decision environments. To demonstrate its feasibility and effectiveness, the model is empirically tested within the domain of teaching effectiveness evaluation, notably for an engineering course, leveraging multi-source data and cross-period observations. Comparative analyses with conventional expert scoring and static weighting methods are conducted to assess decision stability, sensitivity, and robustness.

The remainder of this paper is organized as follows. Section 2 presents the construction of the hierarchical evaluation structure and the formulation of interval-valued intuitionistic fuzzy AHP. Section 3 introduces the entropy-triggered dynamic weight correction mechanism. Section 4 describes the data structure and preprocessing procedures. Section 5 reports the application results and comparative analyses. Finally, Section 6 concludes the paper and discusses potential extensions of the proposed framework.

## 2. Hierarchical Criteria System Construction for MCDM

In complex multi-criteria decision-making (MCDM) problems, evaluation objectives are typically influenced by multiple interrelated criteria with heterogeneous characteristics and uncertain information sources. To ensure interpretability, completeness, and consistency in the subsequent weighting and aggregation processes, it is essential to construct a well-defined hierarchical criteria system that accurately reflects the structural relationships among decision factors.

**Figure 1:**
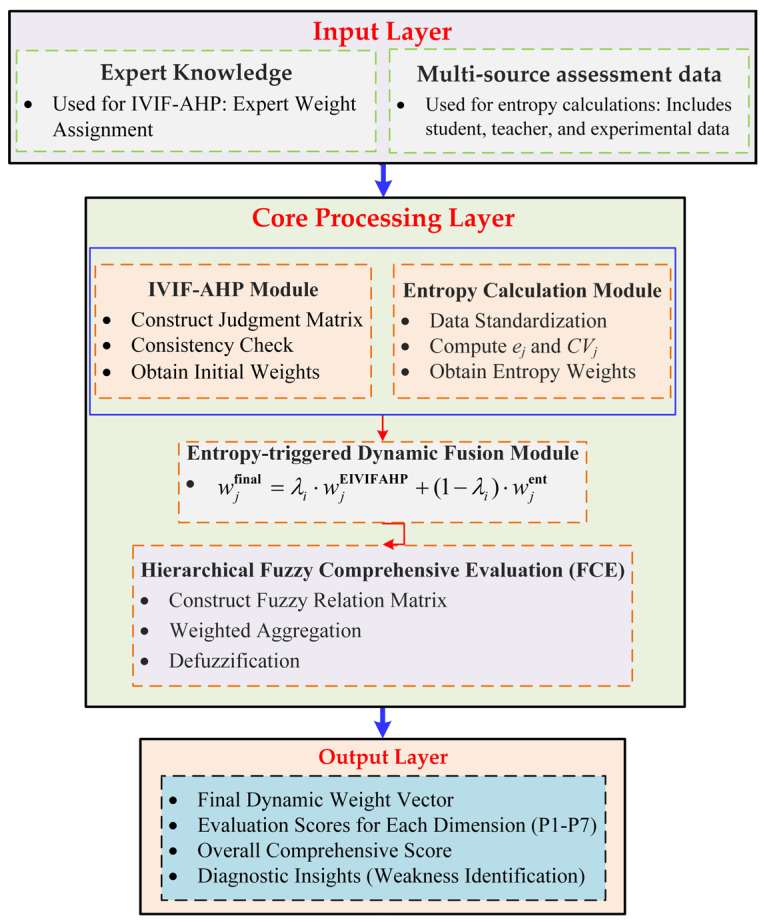
The Proposed Dynamic Hybrid Weighting and Evaluation Framework.

The Dynamic Hybrid Weighting and Evaluation Framework integrates both expert knowledge and multi-source data for dynamic multi-criteria decision-making (MCDM). As shown in Figure 1, the framework consists of three layers: the Input Layer collects expert knowledge (via IVIF-AHP for expert weight assignment) and multi-source data (used for entropy calculations); the Core Processing Layer includes modules for IVIF-AHP, entropy calculation, and a dynamic fusion mechanism that adjusts weights based on both expert judgments and data dispersion; and the Output Layer produces the final dynamic weight vector, evaluation scores for each dimension, an overall comprehensive score, and diagnostic insights to identify areas for improvement. This framework addresses the limitations of static weighting methods by adapting to varying data distributions, ensuring more robust and flexible decision-making outcomes.

### 2.1. Indicator Selection and Quantification Criteria

In this study, a two-level hierarchical criteria system is established to model the considered complex evaluation problem. The first-level criteria represent aggregated decision dimensions that capture the principal aspects of system performance, while the second-level criteria consist of measurable sub-criteria used for quantitative assessment. This hierarchical formulation provides a structured foundation for applying an interval-valued intuitionistic fuzzy analytic hierarchy process (IVIF-AHP) to derive initial subjective weights and for implementing entropy-based dynamic weight correction in the subsequent methodology. We distinguish between criteria as the broad evaluation dimensions (first-level indicators, e.g., P1–P7) and indicators as their specific, measurable sub-components (second-level indicators, e.g., b1–b14). The selection process below focuses on defining the latter to operationalize the former within our hierarchical evaluation framework.

The construction of the criteria system is guided by domain knowledge, expert consultation, and practical constraints of the evaluated system. A structured expert elicitation process is employed to ensure the relevance and rationality of the selected criteria, while statistical consistency measures are used to enhance robustness. The resulting hierarchical criteria system serves as the core decision structure for the proposed dynamic hybrid weighting and fuzzy evaluation framework.

To validate the proposed framework, it is applied to the Building Power Supply and Distribution Technology (BPSDT) course. A dual-dimensional evaluation system integrating student competency and teacher instruction quality is constructed. The design of this system is grounded in engineering education accreditation standards, industry technical specifications, and pedagogical reform objectives.

The indicator selection process adopts the Delphi method [16,17] for multiple rounds of expert consultation and validation. The procedure is summarized as follows:

(1) Expert Selection: Experts were selected based on professional authority, requiring either no less than 10 years of experience in building electrical engineering projects or no less than 8 years of teaching experience in higher education. The expert panel consisted of both academic faculty members and industry practitioners.
(2) Preliminary Indicator Screening: Based on an extensive literature review and curriculum objectives, 14 secondary indicators were initially identified. A five-point Likert scale [18] was employed to assess the importance of each indicator, and experts were encouraged to provide supplementary suggestions.
(3) Consensus Evaluation: Kendall’s coefficient of concordance (W) [19] was used to evaluate the consistency of expert opinions, with a threshold of W ≥ 0.7 indicating high consensus. Indicators with a coefficient of variation (CV) ≤ 0.25 were retained; otherwise, adjustments were made in subsequent rounds.
(4) Finalization of Indicators: Indicators exhibiting persistently low consensus were removed based on expert feedback, resulting in a stable evaluation framework comprising seven first-level indicators and fourteen secondary indicators.

Through this iterative Delphi process, the constructed evaluation system achieves both statistical validity and practical relevance. A critical aspect of this methodology is the clear separation between the indicator screening phase and the weight determination phase. The Likert scale scores collected during the Delphi process were used exclusively in the screening phase to assess indicator importance and to measure expert consensus using Kendall’s W and the coefficient of variation. This phase culminated in the final hierarchical indicator system (Table 1). The subsequent weight determination employs the Interval-Valued Intuitionistic Fuzzy AHP (IVIF-AHP), which operates on this finalized structure using an entirely independent scale—the Interval-Valued Intuitionistic Fuzzy Numbers (IVIFNs) defined in Table 2—to perform pairwise comparisons. Therefore, the Likert scale data are not fed into the IVIF-AHP model as inputs; their role is strictly confined to constructing the model’s framework. The finalized dual-dimensional framework explicitly addresses two core aspects of engineering education, as detailed below.

#### 2.1.1. Student Competency Dimension

This dimension focuses on cultivating engineering practice outcomes and innovation capabilities, and includes three first-level indicators:

(1) Engineering Practice Competency (P1): Evaluates hands-on engineering skills through secondary indicators such as Power Distribution System Debugging (b1) and Lighting Scheme Implementation (b2).
(2) Technical Documentation Competency (P2): Assesses standardized technical communication abilities via indicators including Electrical Design Documentation Standards (b3) and Energy Efficiency Assessment Report (b4).
(3) Collaborative Innovation Competency (P3): Emphasizes interdisciplinary collaboration and system integration capabilities, represented by Smart Construction Collaboration (b5) and Smart Control System Integration (b6).

These indicators are aligned with the competency requirements of the Washington Accord and incorporate current industry standards (e.g., GB55024-2022), ensuring close integration with real engineering demands.

#### 2.1.2. Teacher Instruction Dimension

This dimension concentrates on instructional implementation quality and pedagogical innovation, and includes four first-level indicators:

(1) Engineering Teaching Literacy (P4): Measures the ability to translate engineering experience into effective teaching resources, using Industry Case Updates (b7) and On-site Teaching Capability (b8).
(2) Integration of Technological Frontiers (P5): Evaluates the incorporation of emerging technologies into curricula through Smart Lighting Technology (b9) and Green Building Standards (b10).
(3) Teaching Process Execution (P6): Assesses instructional standardization via Blended Virtual Experiments (b11) and Safety Standard Penetration (b12).
(4) Depth of Industry–Education Interaction (P7): Reflects the effectiveness of university–enterprise collaboration using Corporate Mentor Involvement (b13) and Engineering Ethics Care (b14).

By synthesizing global engineering education evaluation research and the specific characteristics of the Building Electrical and Intelligent Engineering (BEI) major, the proposed two-dimensional indicator system forms a comprehensive teaching quality monitoring framework. The complete hierarchical index system is presented in Table 1.

### 2.2. Enhanced Interval-Valued Intuitionistic Fuzzy AHP Weight Determination

**Figure 2:**
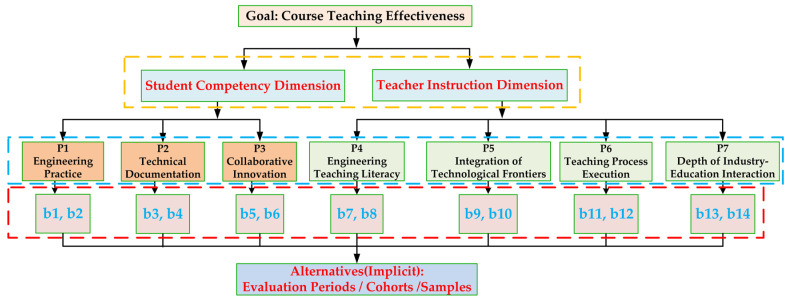
Hierarchical Structure of the IVIF-AHP Model for Course Evaluation.

The IVIF-AHP is applied to the two-level criteria system established in Section 2.1. As illustrated in Figure 2, the hierarchical decision model is structured with the Goal (Comprehensive Evaluation), Criteria (the seven first-level indicators P1–P7), and Sub-Criteria (the fourteen second-level indicators b1–b14). Within this structure, IVIF-AHP requires systematic pairwise comparisons at two levels: (1) among all Criteria relative to the Goal, to determine their global importance; and (2) among the Sub-Criteria within each Criterion group, to determine their local weights. It is important to note that in this dynamic evaluation context, the traditional “Alternatives” are implicit, represented by different evaluation periods, cohorts, or samples (e.g., the n = 127 samples), whose performance is dynamically assessed against this weighted hierarchy.

#### 2.2.1. Theoretical Foundation of Interval-Valued Intuitionistic Fuzzy Sets

In multi-criteria decision-making (MCDM) problems, expert judgments are often accompanied by uncertainty and cognitive hesitation. To precisely model such complex information, this study is grounded in the theory of interval-valued intuitionistic fuzzy sets (IVIFS). IVIFS not only effectively expresses the three psychological states of support, opposition, and hesitation in expert judgments but also provides a theoretical foundation for the subsequent enhanced interval-valued intuitionistic fuzzy AHP (IVIF-AHP) model. This section aims to systematically elaborate on the conceptual evolution, core definitions, and the pivotal role of IVIFS in this framework.

(1) Conceptual Evolution from Fuzzy Sets to Interval-Valued Intuitionistic Fuzzy Sets

The classical mathematical tool for handling uncertainty is the fuzzy set, introduced by Zadeh, which describes the degree to which an element (*x*) belongs to a set *A* using a membership function $\mu_{A}(x)\in [0,1]$, thus modeling the phenomenon of “both this and that” [20]. However, in actual expert decision-making, the expression of a judgment often involves not only the degree of “support” (membership) but also a clear degree of “opposition” (non-membership), as well as “hesitation” due to insufficient information. Traditional fuzzy sets are unable to simultaneously represent these three psychological states.

To overcome this limitation, Atanassov introduced intuitionistic fuzzy sets (IFSs) [21]. In IFSs, each element *x* is assigned both a membership $\mu_{A}(x)$ and a non-membership $\nu_{A}(x)$, subject to the constraint $0\leq \mu_{A}(x)+\nu_{A}(x)\leq 1$ Consequently, the hesitancy degree $\pi_{A}(x)=1-\mu_{A}(x)-\nu_{A}(x)$ is explicitly defined, quantifying the uncertainty and cognitive fuzziness in a judgment. The triplet (μ, ν, π) in IFS provides a richer semantic foundation for describing support, opposition, and hesitation.

However, in group decision-making scenarios, it is often unrealistic to require multiple experts to provide precise values for membership and non-membership; a more natural expression is to provide a confidence interval. Hence, the interval-valued intuitionistic fuzzy set (IVIFS) was introduced, which extends the point values in IFS to interval values [22]. IVIFS uses membership intervals $\left[\mu^{L},\mu^{U}\right]$ and non-membership intervals $\left[\nu^{L},\nu^{U}\right]$ to represent information. This extension not only retains the ability to quantify hesitation but also flexibly accommodates individual expert judgment differences and the fuzziness inherent in the evaluation itself, making it a powerful tool for dealing with higher-order uncertainty.

(2) Core Mathematical Definitions

Based on the above evolution, we provide the strict mathematical definitions adopted in this study.

**Definition 1.**  *Interval-Valued Intuitionistic Fuzzy Number; let X be a non-empty domain. An interval-valued intuitionistic fuzzy set A can be represented as*

(1) $$A=\left\{\left.\langle x,[\mu_{A}^{L}(x),\mu_{A}^{U}(x)],[\nu_{A}^{L}(x),\nu_{A}^{U}(x)]\rangle |x\in X\right\}\right.,$$

*where* $\left[\mu_{A}^{L}(x),\mu_{A}^{U}(x)]\subseteq [0,1\right]$ *and* $\left[\nu_{A}^{L}(x),\nu_{A}^{U}(x)]\subseteq [0,1\right]$ *are referred to as the membership interval and non-membership interval of element x belonging to set A, respectively, and they satisfy the condition*

(2) $$\mu_{A}^{U}(x)+\nu_{A}^{U}(x)\leq 1.$$

*The pair* $\tilde{a}=\langle [\mu^{L},\mu^{U}],[\nu^{L},\nu^{U}]\rangle$ *is called an interval-valued intuitionistic fuzzy number (IVIFN), which is the basic unit for expressing pairwise comparison judgments made by experts in this study.*

**Definition 2.**  *Hesitancy Interval. Based on Definition 1, the hesitancy interval corresponding to element (x) in set (A) is defined as*

(3) $$\pi_{A}(x)=[\pi_{A}^{L}(x),\pi_{A}^{U}(x)]=[1-\mu_{A}^{U}(x)-\nu_{A}^{U}(x),1-\mu_{A}^{L}(x)-\nu_{A}^{L}(x)].$$

*The hesitancy interval intuitively quantifies the range of uncertainty in a judgment, and its width* $\pi_{A}^{U}(x)-\pi_{A}^{L}(x)$ *reflects the degree of cognitive fuzziness.*

The adoption of IVIFN in this framework is motivated by the goal of enabling a data-informed dynamic fusion. IVIFN achieves this by formally quantifying expert hesitation through the hesitancy interval. This interval provides a direct, quantitative measure of consensus among experts for each indicator. In the subsequent mechanism, this subjective measure of uncertainty is directly compared with the objective data dispersion for the same indicator, creating a coherent rationale for dynamically adjusting the influence of expert weights versus data-driven weights.

#### 2.2.2. Expert Judgment Modeling and IVIFN Scale

Building on the theoretical foundation established in Section 2.2.1, this section explains how the interval-valued intuitionistic fuzzy numbers (IVIFNs) are applied to model expert judgments [23,24].

(1) Expert Judgment Matrix Construction

Experts meeting the criteria outlined in Section 2.1 were invited to participate in the weighting process. Each expert compared indicators within the same level pairwise. Their judgments were quantified using an interval-valued intuitionistic fuzzy judgment matrix:

(4) $${\tilde{A}}^{\left(e\right)}={\left[{\tilde{a}}_{ij}^{\left(e\right)}\right]}_{n\times n},$$

where each element ${\tilde{a}}_{ij}^{\left(e\right)}$ represents the IVIFN corresponding to expert *e*’s judgment on the importance of indicator *i* relative to indicator *j*. The matrix satisfies the reciprocal condition, meaning

(5) $${\tilde{a}}_{ji}^{\left(e\right)}={\left({\tilde{a}}_{ij}^{\left(e\right)}\right)}^{-1},$$

where the reciprocal operation ${\left(\cdot \right)}^{-1}$ is defined as swapping the membership and non-membership intervals of the IVIFN, i.e., if $\tilde{a}=\langle [\mu^{L},\mu^{U}],[\nu^{L},\nu^{U}]\rangle$, then ${\tilde{a}}^{-1}=\langle [\nu^{L},\nu^{U}],[\mu^{L},\mu^{U}]\rangle$. This is consistent with the reciprocal scales provided in Table 2.

(2) Operationalization Using the IVIFN Semantic Scale

To standardize the conversion of linguistic judgments into IVIFNs, this study employs a dedicated 9-level semantic scale, as defined in Table 2. This use of IVIFNs constitutes the fuzzification step, translating linguistic expert judgments into a formal fuzzy set representation for processing. Experts select a linguistic term that corresponds to a predefined IVIFN.

The design of this scale follows a cognitive logic: as the importance level increases, the membership interval $\left[\mu^{L},\mu^{U}\right]$ shifts upward while the non-membership interval $\left[\nu^{L},\nu^{U}\right]$ shifts downward. Correspondingly, the hesitancy interval $\left[\pi^{L},\pi^{U}\right]$ reflects the inherent uncertainty at each level—broader for moderate judgments and narrower for extreme ones, aligning with the psychology of decision-making where stronger convictions are typically held with greater certainty.

(3) Output for Subsequent Processing

The outcome of this step is a set of *k* individual IVIFN judgment matrices $\left\{{\tilde{A}}^{\left(1\right)},\ldots ,{\tilde{A}}^{\left(k\right)}\right\}$. These matrices, which encapsulate both the preference intensity and associated uncertainty of each expert, serve as the direct input for the group opinion aggregation in Section 2.2.3.

#### 2.2.3. Aggregation of Group Opinions

To synthesize the judgment matrices ${\tilde{A}}^{\left(e\right)}$ from *k* experts, an enhanced weighted arithmetic averaging (WAA) operator is employed. Unlike traditional aggregation methods, this operator integrates both expert credibility and assessment similarity to achieve refined weight adjustment [25,26]:

(6) $${\tilde{a}}_{ij}=\langle \left[1-\prod_{e=1}^{k}{\left(1-\mu_{ij}^{L(e)}\right)}^{w_{e}},1-\prod_{e=1}^{k}{\left(1-\mu_{ij}^{U(e)}\right)}^{w_{e}}\right],\left[\prod_{e=1}^{k}{\left(\nu_{ij}^{L(e)}\right)}^{w_{e}},\prod_{e=1}^{k}{\left(\nu_{ij}^{U(e)}\right)}^{w_{e}}\right]\rangle ,$$

The expert weight *w_e_* is obtained by normalizing the credibility measure *C_e_*:

(7) $$w_{e}=\frac{C_{e}}{\sum_{e=1}^{k}C_{e}},$$

where expert credibility is assigned according to professional background: professor/professor-level senior engineer (0.9), associate professor/senior engineer (0.8), and lecturer/engineer (0.7).

To further mitigate individual subjectivity, an expert assessment similarity measure $S_{ij}^{\left(e\right)}$ is introduced:

(8) $$S_{ij}^{\left(e\right)}=1-\frac{1}{4}\left(\begin{array}{l} \left|\mu_{ij}^{L(e)}-{\bar{\mu }}_{ij}^{L}|+|\mu_{ij}^{U(e)}-{\bar{\mu }}_{ij}^{U}\right|\\ +|\nu_{ij}^{L(e)}-{\bar{\nu }}_{ij}^{L}|+|\nu_{ij}^{U(e)}-{\bar{\nu }}_{ij}^{U}| \end{array}\right),$$

where the group mean membership and non-membership degrees are given by

(9) $$\left\{\begin{array}{l} {\bar{\mu }}_{ij}^{L}=\frac{1}{k}\sum_{e=1}^{k}\mu_{ij}^{L(e)}\\ {\bar{\mu }}_{ij}^{U}=\frac{1}{k}\sum_{e=1}^{k}\mu_{ij}^{U(e)} \end{array}\right.,$$

(10) $$\left\{\begin{array}{l} {\bar{\nu }}_{ij}^{L}=\frac{1}{k}\sum_{e=1}^{k}\nu_{ij}^{L(e)}\\ {\bar{\nu }}_{ij}^{U}=\frac{1}{k}\sum_{e=1}^{k}\nu_{ij}^{U(e)} \end{array}\right..$$

The adjusted expert weight $w_{e}^{*}$ is then calculated as

(11) $$w_{e}^{*}=\lambda w_{e}+(1-\lambda )\cdot S_{ij}^{\left(e\right)}\;\;\;\;(\lambda =0.6),$$

#### 2.2.4. Defuzzification and Consistency Verification

To obtain precise numerical values, IVIFNs are transformed using the *α*-cut method [23]:

(12) $$a_{ij}^{\alpha }=\frac{1}{2}\left[\alpha \cdot \left(\mu_{ij}^{L}+\mu_{ij}^{U}\right)+(1-\alpha )\cdot \left(1-\nu_{ij}^{U}-\nu_{ij}^{L}\right)\right],$$

where α ∈ [0,1] balances the influence of membership and non-membership degrees. In this study, *α* = 0.5 is adopted to maintain a neutral decision stance. This *α*-cut operation serves as the defuzzification step, converting the interval-valued fuzzy weights into crisp values for subsequent integration with the objective entropy weights.

The maximum eigenvalue *λ*_max_ and the corresponding eigenvector **W** of the judgment matrix are obtained by

(13) $$A^{\alpha }W=\lambda_{\max}W.$$

Consistency of the judgment matrix is verified using the consistency ratio (CR) [1]:

(14) $$CR=\frac{CI}{RI}=\frac{\lambda_{\max}-n}{(n-1)RI},$$

If *CR* < 0.1, the judgment matrix is considered consistent; otherwise, expert evaluations must be revised.

The enhanced IVIF-AHP framework integrates credibility-adjusted group consensus and α-cut-based defuzzification, thereby achieving robust handling of uncertainty and expert disagreement. While the initial weights obtained from IVIF-AHP effectively capture subjective judgment uncertainty, they remain inherently subjective. To address this limitation, the subsequent section introduces an entropy-based dynamic correction mechanism that objectively reflects indicator importance using real instructional data.

## 3. Design of a Dynamic Entropy-Triggered Weight Correction Mechanism

To address the rigidity of static hybrid weighting, this section proposes a dynamic entropy-triggered weight correction mechanism that combines objective data dispersion with subjective IVIF-AHP weights. It adapts the fusion ratio between subjective and objective weights by using information entropy and the coefficient of variation to regulate indicator sensitivity.

### 3.1. Data Standardization and Information Entropy Analysis

Let *n* denote the number of evaluation samples and *m* = 14 the number of secondary indicators. The original evaluation data matrix is denoted by $R={\left[r_{ij}\right]}_{n\times m}$, where $r_{ij}$ represents the score of the *i*-th sample with respect to indicator *j*.

To eliminate dimensional inconsistency and ensure comparability among indicators, min–max normalization is applied:

(15) $$r_{ij}^{\mathrm{std}}=\frac{r_{ij}-\min(r_{j})}{\max(r_{j})-\min(r_{j})},$$

where max(*r_j_*) and min(*r_j_*) denote the maximum and minimum values of indicator *j*, respectively.

Information entropy, originally introduced by Shannon, is employed to quantify the uncertainty and dispersion of standardized evaluation data [27]. For indicator *j*, the entropy value *e_j_* is defined as

(16) $$e_{j}=-\frac{1}{\ln n}\sum_{i=1}^{n}p_{ij}\ln p_{ij},$$

where

(17) $$p_{ij}=\frac{r_{ij}^{\mathrm{std}}}{\sum_{i=1}^{n}r_{ij}^{\mathrm{std}}}$$

denotes the normalized contribution of the *i*-th sample to indicator *j*.

Based on the entropy values, the objective entropy weight of indicator *j* is computed as

(18) $$w_{j}^{\mathrm{ent}}=\frac{1-e_{j}}{\sum_{k=1}^{m}\left(1-e_{k}\right)}\;\;\;,$$

where 1 − *e_j_* is termed the deviation coefficient, which measures the effective information content of indicator *j*. A smaller entropy *e_j_* results in a larger deviation coefficient, thereby assigning a greater weight to that indicator in the objective weighting scheme. The denominator performs normalization, ensuring that all entropy weights sum to one $\sum_{j=1}^{m}w_{j}^{\mathrm{ent}}=1$.

To further characterize relative variability, the coefficient of variation is introduced:

(19) $$CV_{j}=\frac{\sigma_{j}}{{\bar{x}}_{j}},$$

where $\sigma_{j}$ and ${\bar{x}}_{j}$ denote the standard deviation and mean of indicator *j*, respectively.

By jointly considering *e_j_* and *CV_j_*, indicators are classified according to their sensitivity characteristics:

(1) When *e_j_* > 0.7 and *CV_j_* > 0.3, it is identified as a high-dispersion indicator;
(2) When *e_j_* < 0.3 and *CV_j_* < 0.1, it is identified as a low-controversy indicator.

The classification thresholds for *e_j_* and *CV_j_* are adopted based on established conventions in information theory and statistics for distinguishing between low, moderate, and high levels of dispersion and consensus [28,29]. This classification provides a quantitative basis for adaptive weight regulation.

### 3.2. Entropy-Triggered Dynamic Weight Fusion

Let $w_{j}^{\mathrm{EIVIFAHP}}$ denote the initial subjective weight obtained from the enhanced IVIF-AHP procedure, and $w_{j}^{\mathrm{ent}}$ the objective entropy-based weight. The final weight of indicator *j* is defined as a convex combination:

(20) $$w_{j}^{\mathrm{final}}=\beta \cdot w_{j}^{\mathrm{EIVIFAHP}}+(1-\beta )\cdot w_{j}^{\mathrm{ent}},$$

where β∈[0, 1] is an adaptive fusion coefficient.

(21) $$\beta =\left\{\begin{array}{ll} 0.7 & e_{j}<0.3\;\mathrm{and}\;CV_{j}<0.1\;\\ 0.5 & 0.3\leq e_{j}\leq 0.7\;\mathrm{and}\;0.1\leq CV_{j}\leq 0.3\\ 0.3 & e_{j}>0.7\;\mathrm{and}\;CV_{j}>0.3 \end{array}\right..$$

This piecewise definition ensures that indicators with low dispersion and high expert consensus preserve the dominance of subjective knowledge, whereas indicators exhibiting high variability are more strongly influenced by data-driven information.

The proposed entropy-triggered fusion mechanism constitutes a bounded and adaptive weighting strategy, effectively balancing expert judgment and empirical evidence. By dynamically regulating weight contributions at the indicator level, the method mitigates the limitations of static entropy-based or expert-driven approaches and establishes a mathematically consistent foundation for subsequent fuzzy comprehensive evaluation.

## 4. Data Collection and Preprocessing

This section describes the data structure and preprocessing procedures adopted to support the proposed dynamic hybrid weighting and fuzzy evaluation framework. The objective is to construct a reliable and consistent input data matrix for entropy-based weight correction and subsequent fuzzy comprehensive evaluation under a multi-source decision environment.

### 4.1. Multi-Source Data Collection Framework

To ensure robustness and information completeness, a multi-source data integration strategy based on the triangulation principle is adopted. Evaluation data are collected from heterogeneous sources corresponding to different aspects of the decision system, thereby forming an evidence-based representation of indicator performance across the entire evaluation process.

The data collection framework consists of four categories: subjective assessment data, objective performance records, process-related documentation, and expert observational evaluations. Each data category is mapped to corresponding secondary indicators to ensure structural consistency within the hierarchical criteria system. The overall framework, including acquisition methods, sample sizes, and indicator coverage, is summarized in Table 3.

### 4.2. Sample Composition and Timeline

The evaluation dataset consists of two complementary components: sample-based assessment data and expert judgment data.

(1) Sample-based data were collected from n = 127 valid samples, representing multiple evaluation stages within the considered decision period. These data provide quantitative measurements for secondary indicators derived from questionnaires, experimental records, and platform logs.
(2) Expert judgment data were obtained from *k* = 10 domain experts selected according to predefined experience criteria. The expert panel comprises both academic specialists and industry practitioners to ensure balanced coverage of theoretical and practical perspectives.

Data acquisition was conducted over a complete evaluation cycle and organized into three representative stages: initial assessment, intermediate evaluation, and final assessment. This temporal structure enables the capture of indicator performance variations and supports cross-stage aggregation in subsequent analysis.

### 4.3. Data Preprocessing and Standardization Procedures

Given the heterogeneous nature of the collected data, a hierarchical preprocessing strategy is employed to ensure consistency, comparability, and numerical stability.

(1) Subjective Assessment Data

Subjective evaluation data obtained from questionnaires are first processed to ensure directional consistency. Negatively worded items are reverse-coded to align evaluation polarity across indicators. Missing values are handled using the K-nearest neighbors (KNNs) imputation method with *k* = 5, which preserves local data structure while minimizing distortion [30].

(2) Objective Performance Data

Objective data derived from experimental records and platform logs are screened for outliers using the boxplot method. Detected outliers account for approximately 1.7% of the total observations and are corrected to prevent disproportionate influence on entropy calculations.

All indicators are subsequently normalized to the interval [0,1] using the min–max range transformation defined in Section 3, thereby eliminating scale effects and ensuring compatibility with entropy-based weighting.

## 5. Model Implementation and Computational Outcomes

This section reports the procedural execution and direct numerical outputs of the proposed entropy-triggered IVIF-AHP–FCE framework, focusing on the generation of weights, scores, and intermediate results.

### 5.1. Generation of Initial Weights via Enhanced IVIF-AHP

A group of ten experts was invited to construct the interval-valued intuitionistic fuzzy judgment matrices. The expert group comprised academic specialists and industry practitioners, ensuring coverage of both theoretical and practical perspectives.

**Figure 3:**
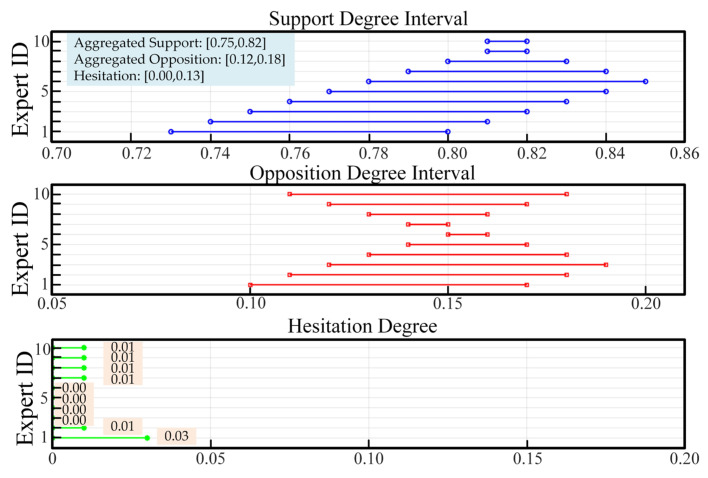
IVIFN Interval Distributions of Indicator P1 by 10 Experts.

For each first-level indicator *P_i_* (i = 1, …, 7), interval-valued intuitionistic fuzzy numbers were used to represent pairwise comparisons. As an illustrative example, for indicator *P*_1_, the aggregated membership interval was [0.75,0.82], the non-membership interval [0.12,0.18], and the hesitancy interval [0.00,0.13], indicating moderate expert consensus with bounded uncertainty. Figure 3 illustrates the IVIFN interval distributions for Engineering Practice Competency (P1) by the 10 experts. The same enhanced IVIF-AHP process was applied to other first-level indicators (P2–P7), including matrix construction, WAA aggregation, and defuzzification.

The group judgment matrices were aggregated using the weighted arithmetic averaging operator, followed by credibility-adjusted weighting and α-cut defuzzification with α = 0.5. Consistency verification confirmed that all judgment matrices satisfied the standard AHP consistency requirement.

The resulting initial weight vector obtained from the enhanced IVIF-AHP procedure is ***W***^EIVIF-AHP^ = [0.291,0.203,0.118,0.109,0.087,0.132,0.060]^T^. Table 4 details the weights and engineering significance of each indicator.

### 5.2. Entropy-Triggered Dynamic Weight Adjustment

Based on the standardized data matrix, information entropy values were computed for each indicator using Equations (14)–(16). The entropy-trigger mechanism defined in Section 3 was then applied to dynamically regulate the fusion coefficient *β* at the indicator level.

**Figure 4:**
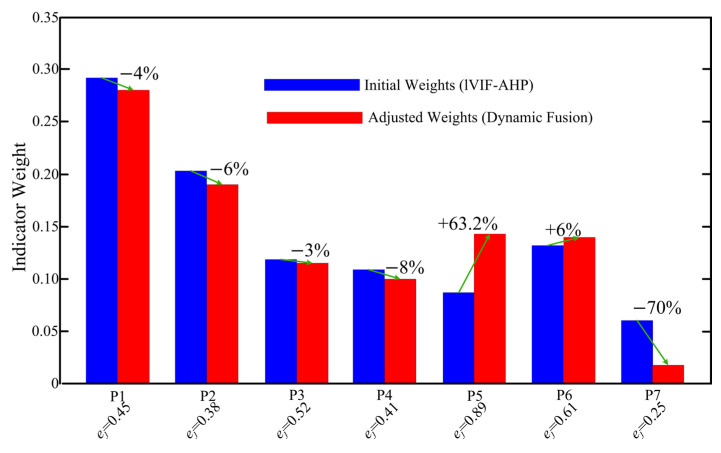
Comparison of Indicator Weights Before and After Entropy Weight Correction.

Indicators exhibiting high entropy and large coefficients of variation received increased objective weight contributions, while indicators with low dispersion retained dominance of expert-derived weights. Figure 4 illustrates the comparison between initial and adjusted weights.

After entropy-triggered fusion, the final weight vector is obtained as

**W***_adjusted_* = [0.284,0.193,0.117,0.102,0.144,0.142,0.018]^T^.

### 5.3. Hierarchical Fuzzy Comprehensive Evaluation

This subsection implements the hierarchical fuzzy comprehensive evaluation (FCE) using the final weights obtained in Section 5.2. The objective is to generate grade-membership vectors at the first-level criteria and an overall evaluation vector at the system level, followed by numerical defuzzification.

#### 5.3.1. Evaluation Set and Grade Quantification

Let the evaluation grade set be *E* = {*E*_1_,*E*_2_,*E*_3_,*E*_4_,*E*_5_}, and associate it with a numerical score vector E = {95,85,75,60,30}, consistent with the grading rules summarized in Table 5.

#### 5.3.2. Membership Estimation and Fuzzy Relation Matrices

For each secondary indicator *b_j_* (*j* = 1, …, 14), the grade-membership vector is estimated from frequency statistics over *N* valid samples:

(22) $$r_{jk}=\frac{N_{jk}}{N},$$

To quantify sampling uncertainty, the (normal-approximation) confidence interval is computed as

(23) $$r_{jk}\pm 1.96\sqrt{\frac{r_{jk}(1-r_{jk})}{N}},$$

The membership vectors are grouped according to their parent first-level criterion *P_i_*. Stacking the membership vectors of the *m_i_* subordinate indicators forms the fuzzy relation matrix

(24) $$R_{Pi}\in R^{m_{i}\times 5}.$$

#### 5.3.3. Two-Level Fuzzy Synthesis and Defuzzification

Let $W_{b_{i}}\in R^{m_{i}}$ denote the normalized weight vector of secondary indicators under *P_i_* (obtained after entropy-triggered correction and normalization). The first-level fuzzy evaluation vector is computed by the weighted-average synthesis operator

(25) $$B_{i}=W_{b_{i}}^{T}\times R_{P_{i}},$$

Stacking ${\left\{B_{i}\right\}}_{i=1}^{7}$ row-wise yields the first-level evaluation matrix

(26) $$B=\left[\begin{array}{c} B_{1}\\ B_{2}\\ \vdots \\ B_{7} \end{array}\right]=\left[\begin{array}{ccccc} r_{1,\;E1} & r_{1,\;E2} & r_{1,\;E3} & r_{1,\;E4} & r_{1,\;E5}\\ r_{2,\;E1} & r_{2,\;E2} & r_{2,\;E3} & r_{2,\;E4} & r_{2,\;E5}\\ \vdots & \vdots & \vdots & \vdots & \vdots \\ r_{7,\;E1} & r_{7,\;E2} & r_{7,\;E3} & r_{7,\;E4} & r_{7,\;E5} \end{array}\right],$$

Using the entropy-triggered final weight vector $W_{\mathrm{adjusted}}\in R^{7}$ from Section 5.2, the overall fuzzy evaluation vector is obtained as

(27) $$K=W_{adjusted}\times B=[k_{1},k_{2},k_{3},k_{4},k_{5}],$$

Finally, the overall numerical score is computed via defuzzification:

(28) $$S=K\times E^{T}.$$

In the numerical implementation, the resulting comprehensive score is *S* = 90.38, and the corresponding membership vector **K** exhibits dominant mass on the higher-grade components, which is consistent with the obtained score.

The implementation of the proposed framework, as detailed in this section, yields two primary sets of computational outputs that serve as the basis for subsequent analysis. First, it produces the final dynamic weight vector, which results from the entropy-triggered fusion of initial IVIF-AHP weights and objective entropy weights. Second, it generates the hierarchical evaluation results, culminating in an overall comprehensive score and the associated membership distributions. These outputs demonstrate the procedural execution of the framework. The following section will analyze these results in depth, evaluating their comparative performance, practical validation, and underlying mechanisms.

### 5.4. Computational Results, Comparative Behavior, and Robustness Discussion

This subsection analyzes the computational behavior of the proposed entropy-triggered IVIF-AHP–FCE framework from the perspectives of (i) weight redistribution patterns, (ii) evaluation output sensitivity, and (iii) comparative behavior relative to static weighting baselines.

#### 5.4.1. Weight Redistribution Under Entropy Triggering

Let ***W***^EIVIF-AHP^ denote the initial subjective weight vector and ***W***_adjusted_ the final entropy-triggered vector. The adjustment produces a nonuniform redistribution across criteria, reflecting heterogeneous dispersion characteristics in the underlying data. In particular, criteria associated with higher entropy and larger coefficients of variation receive increased objective contributions (smaller *β*), while low-dispersion criteria retain stronger dominance of expert-derived weights (larger *β*). This confirms that the fusion mechanism behaves as a bounded convex regulator rather than a uniform linear correction.

#### 5.4.2. Output Sensitivity and Stability

The hierarchical FCE defines a continuous aggregation pipeline in which **W**_adjusted_ determines the overall membership vector **K**, and **K** is then defuzzified to the scalar score *S*. Since both the fusion step and the fuzzy synthesis step are convex combinations, the overall procedure is numerically stable with respect to bounded perturbations in membership frequencies and weight vectors. Empirically, the obtained overall score *S* = 90.38 results from the concentration of **K** on higher grades, indicating that the final output is not driven by a single criterion but by the aggregated membership distribution under the corrected weights.

#### 5.4.3. Comparative Behavior Against Static Baselines

Compared with conventional expert-only scoring, the proposed framework yields a more conservative and discriminative result by explicitly incorporating (i) expert hesitation via interval-valued intuitionistic fuzzy numbers and (ii) dispersion-aware objective correction via entropy triggering. The reduction of “evaluation inflation” observed in the baseline comparison is consistent with the fact that expert-only aggregation lacks a data-dependent mechanism for suppressing over-confident weights under low-information or high-uncertainty regimes. In contrast, the proposed mechanism adaptively reallocates importance when dispersion signals indicate heterogeneous performance, leading to improved diagnostic resolution in the aggregated outcome.

## 6. Results and Discussion

Building upon the computational results presented in Section 5, this section provides a comprehensive analysis, validation, and interpretation. We evaluate the framework’s performance, examine its empirical effectiveness, explain its internal mechanisms, and discuss its generalizability.

### 6.1. Comparative Performance and Diagnostic Precision

This analysis evaluates the framework’s output against conventional methods. The direct comparison, as summarized in Table 6, reveals key differences in scoring and uncertainty handling.

The proposed model yields a comprehensive score of 90.38, which is 3.87 points (4.1%) lower than the score from the traditional expert-only method (94.25). This difference constitutes a correction of “evaluation inflation,” a common pitfall in purely subjective methods where expert optimism, unchecked by data variability, leads to overestimation. The most pronounced correction is observed for the “Depth of Industry–Education Interaction (P7),” where our model’s score (76.2) is 12.5 points lower. This result is directly attributable to the entropy-triggered mechanism: P7 exhibited both high expert hesitancy and high data dispersion (*e_j_* > 0.7, *CV_j_* > 0.3), causing a significant reduction in its subjective weight influence. Thus, Table 6 validates the model’s core capability to uncover latent weaknesses by integrating objective data dispersion with subjective judgments.

### 6.2. Validation Through Intervention and Measurable Improvement

This analysis presents empirical evidence of the framework’s practical utility. Guided by the model’s diagnostic output, which identified three underperforming indicators (b9, b14, b7), targeted pedagogical interventions were implemented. The efficacy of these interventions is rigorously validated by the longitudinal data presented in Table 7.

As Table 7 shows, all three indicators demonstrate statistically significant improvements (*p* < 0.05). Beyond score increases, a critical outcome is the reduction in performance dispersion. For b9, the standard deviation *σ* decreased from 12.3 to 8.7—a 29% reduction—signifying a more uniform understanding among students. This full-cycle validation process (diagnosis → intervention → confirmed improvement) establishes the framework as an effective tool for evidence-based instructional design.

### 6.3. Comprehensive Discussion: Mechanism, Robustness, and Generalizability

This section synthesizes an in-depth discussion on the proposed framework, moving beyond specific results to examine its intrinsic operational mechanism, inherent stability, and broader methodological value.

(1) Operational Mechanism of the Dynamic Fusion

The framework’s ability to produce differentiated results, as seen in Section 6.1 and Section 6.2, is rooted in its core fusion mechanism. It successfully establishes distinct weighting regimes: indicators with high information entropy and large coefficients of variation receive increased objective weight contributions (smaller λ), while those with low dispersion retain the dominance of expert-derived weights (larger λ). This adaptive behavior, governed by the decision rules in Equation (19), confirms that the entropy-trigger acts as a bounded, convex regulator. This design ensures a principled balance between data-driven evidence and expert knowledge, leading to smoother weight transitions and enhanced discriminative power across heterogeneous criteria, rather than applying a uniform linear correction.

(2) Inherent Stability and Robustness

The reliability of this adaptive mechanism under varying conditions is a key strength. The consistency of weight adjustment patterns across different evaluation periods confirms the stability of the entropy-triggered logic. Indicators with persistently high dispersion consistently exert greater influence, while stable indicators show minimal weight fluctuation. Furthermore, the observed reduction in evaluation variance across periods demonstrates the framework’s capacity to suppress noise-induced oscillations. This robustness is mathematically inherent to the design, arising from the bounded convex fusion of subjective and objective weights, which guarantees numerical stability and prevents abrupt, unwarranted shifts in decision outcomes amidst data uncertainty.

(3) Methodological Contributions and Generalizability

Synthesizing the above analyses, the broader contribution of the framework lies in its modular and theory-grounded architecture, which effectively reconciles expert uncertainty with data variability within a unified, adaptive MCDM weighting mechanism. Its success stems from the synergistic integration of three components: IVIF-AHP captures subjective hesitation, entropy/CV quantifies objective dispersion, and a rule-based fusion dynamically balances them. This design is intentionally domain-agnostic. Its pilot application in a different engineering course (Building Information Facility Systems) confirmed its transferable capability to enhance diagnostic accuracy and guide interventions. Therefore, the proposed framework transcends a case-specific solution; it offers a replicable paradigm for dynamic evaluation. It bridges the gap between expert-driven “knowledge” and data-driven “evidence,” presenting strong potential for extension to other complex decision domains such as risk assessment, performance evaluation, and decision-support systems characterized by uncertain judgments and heterogeneous data sources.

## 7. Conclusions

This paper proposes an entropy-triggered hybrid MCDM framework that integrates an enhanced interval-valued intuitionistic fuzzy analytic hierarchy process (IVIF-AHP) with dynamic entropy-weighted fuzzy comprehensive evaluation, aimed at addressing teaching effectiveness evaluation. The proposed method addresses fundamental limitations of conventional static weighting approaches, including rigidity of indicator importance, insufficient handling of expert uncertainty, and limited sensitivity to heterogeneous data distributions.

The main methodological contributions, developed and validated within the teaching evaluation context, can be summarized as follows:

(1) A hierarchical decision framework was constructed in which expert judgments are modeled using interval-valued intuitionistic fuzzy numbers, enabling explicit representation of support, opposition, and hesitation in pairwise comparisons.
(2) An entropy-triggered correction mechanism was introduced to dynamically regulate the fusion ratio between subjective and objective weights at the indicator level, allowing the evaluation model to adaptively respond to actual dispersion in teaching and learning data while maintaining numerical stability.
(3) A hierarchical fuzzy aggregation process was employed to ensure consistent and interpretable information synthesis across multiple decision layers. The resulting aggregation pipeline exhibits robustness with respect to bounded perturbations in both weight vectors and membership distributions.

By combining expert-driven modeling with data-driven entropy correction under a unified mathematical structure, the proposed framework provides a robust and adaptive solution for teaching effectiveness evaluation under uncertainty. The case study on the BPSDT course demonstrates its practical utility in generating diagnostic insights and supporting evidence-based instructional improvement. Future work may explore theoretical properties of the proposed fusion mechanism and extend the framework to large-scale or real-time decision environments.

## Figures and Tables

**Table 1:** Dual-Dimensional Course Evaluation Index System.

Dimension	First-Level Indicators	Secondary Indicators	Engineering Competency Observation Points	Data Sources
Student	Engineering Practice Competency (P1)	Power Distribution System Debugging (b1)	Transformer Selection Accuracy, Short-Circuit Current Calculation Compliance Rate, Feasibility of Relay Protection Scheme	Lab Report/Simulation Results
		Lighting Scheme Implementation (b2)	Illuminance Simulation Compliance Rate (GB50034-2024), Effectiveness of Energy-Saving Control Strategy, Rationality of Emergency Lighting System Configuration	Design Drawings/Energy Consumption Data
	Technical Documentation Competency (P2)	Electrical Design Documentation Standards (b3)	GB/T50786 Drafting Standard Coverage, Completeness of Design Description, Logical Rigor of Calculation Sheets	Coursework/Industry Mentor Evaluation
		Energy Efficiency Assessment Report (b4)	Accuracy of LPD (Lighting Power Density) Calculation, Proportion of Renewable Energy Solutions, Depth of Economic Analysis	Project Report
	Collaborative Innovation Competency (P3)	Smart Construction Collaboration (b5)	CAD Model Conflict Resolution Rate, Team Task Contribution, Completeness of Collaboration Platform Logs	Collaboration Platform Data
		Smart Control System Integration (b6)	Success Rate of Lighting-AC Joint Debugging, IoT Device Communication Stability, Fault Diagnosis Response Speed	Training Platform Records
Teacher	Engineering Teaching Literacy (P4)	Industry Case Updates (b7)	Proportion of annual real engineering cases, penetration rate of latest corporate standards	Syllabus/Courseware
		On-site Teaching Capability (b8)	Frequency of substation field teaching, completeness of equipment operation demonstrations	Teaching logs/Student feedback
	Integration of Technological Frontiers (P5)	Smart Lighting Technology (b9)	Proportion of class hours on new technologies like LiFi/visible light communication, depth of intelligent control algorithms	Lesson plan review
		Green Building Standards (b10)	Integration of LEED/green building evaluation standards, weight of carbon-neutral design solutions	Course project analysis
	Teaching Process Execution (P6)	Blended Virtual Experiments (b11)	Number of digital twin simulation experiments, compliance rate of physical equipment operation safety standards	Experiment records/Monitoring videos
		Safety Standard Penetration (b12)	Coverage rate of electric shock first aid drills, pass rate of high-voltage operation procedure assessments	Assessment results
	Depth of Industry-Education Interaction (P7)	Corporate Mentor Involvement (b13)	Class hours taught by industry experts, number of real projects converted into teaching cases	Corporate teaching records/Project certifications
		Engineering Ethics Care (b14)	Intensity of safety standard awareness training (frequency of accident case analysis), assessment of professional responsibility	Ethics tests/Behavioral observations

**Table 2:** Complete scale of interval-valued intuitionistic fuzzy numbers.

Scale Level	Scale Meaning	Interval-Valued Intuitionistic Fuzzy Number	Hesitancy Interval	Reciprocal Scale
1	Equally Important	([0.50,0.50], [0.50,0.50])	[0.00,0.00]	([0.50,0.50], [0.50,0.50])
2	Between 1~3	([0.35,0.45], [0.30,0.50])	[0.05,0.20]	([0.30,0.50], [0.35,0.45])
3	Slightly Important	([0.55,0.65], [0.20,0.30])	[0.05,0.25]	([0.20,0.30], [0.55,0.65])
4	Between 3~5	([0.60,0.70], [0.15,0.25])	[0.05,0.25]	([0.15,0.25], [0.60,0.70])
5	Moderately Important	([0.70,0.80], [0.10,0.20])	[0.00,0.20]	([0.10,0.20], [0.70,0.80])
6	Between 5~7	([0.75,0.85], [0.05,0.15])	[0.00,0.20]	([0.05,0.15], [0.75,0.85])
7	Strongly Important	([0.80,0.90], [0.05,0.10])	[0.00,0.15]	([0.05,0.10], [0.80,0.90])
8	Between 7~9	([0.85,0.95], [0.02,0.05])	[0.00,0.13]	([0.02,0.05], [0.85,0.95])
9	Extremely Important	([0.90,0.95], [0.00,0.05])	[0.00,0.10]	([0.00,0.05], [0.90,0.95])

**Table 3:** Data Collection Framework for Teaching Effectiveness Evaluation.

Data Category	Acquisition Method	Sample Size	Secondary Indicators Covered	Data Standardization Method
Student subjective evaluation	Anonymous electronic questionnaire	Valid questionnaires	b3, b5, b9, b14	Range Method and Reverse Scoring Correction
Objective competency indicators	Experiment reports/Collaboration platform logs	127 reports/logs	b1, b2, b6, b11, b12	Boxplot Outlier Removal
Teaching process records	Teaching archives/Industry cooperation certification	Full-semester materials	b7, b8, b10, b13	Manual Review and Cross-Verification
Expert observational assessment	Industry expert scoring sheets	10 experts	All 14 secondary indicators	EIVIF-AHP Aggregation

**Table 4:** Initial Weights of First-level Indicators.

First-Level Indicators	Weight	Rank	Engineering Significance
Engineering Practice Competency (P1)	0.291	1	Core Skills Have the Highest Weight
Technical Documentation Competency (P2)	0.203	2	Standardized Expression Plays a Key Role
Collaborative Innovation Competency (P3)	0.118	5	Reflects the Importance of Teamwork
Engineering Teaching Literacy (P4)	0.109	6	Foundation of Teacher’s Industry Experience
Integration of Technological Frontiers (P5)	0.087	7	New Technology Penetration Needs Strengthening
Teaching Process Execution (P6)	0.132	3	Teaching Implementation Process Carries Weight
Depth of Industry–Education Interaction (P7)	0.060	4	Enterprise Participation Needs Improvement

**Table 5:** Five-Level Evaluation Criteria.

Level	Score Range	Assigned Value	Engineering Competency Mapping
E1 (Excellent)	90–100	95	Fully meets industry standards
E2 (Good)	80–89	85	Meets core requirements
E3 (Medium)	70–79	75	Partial optimization needed
E4 (Pass)	50–69	60	Meets the minimum teaching objectives
E5 (Fail)	0–49	30	Fails educational quality requirements

**Table 6:** Model Comparison: Proposed Framework vs. Traditional Expert Scoring.

Model	Comprehensive Score	Depth of Industry-Academia Interaction (P7) Score	Uncertainty Quantification Capability
EIVIF-AHP and EWFCE model	90.38	76.2	Supports hesitation degree calculation
Traditional Expert Scoring Method	94.25	88.7	Ignores expert disagreements

**Table 7:** Improvement of Key Indicators After Targeted Interventions.

Indicator	2022 Score	2023 Score	Δ (95% *CI*)	*p*-Value
Smart Lighting Tech (b9)	78.5 ± 12.3	86.2 ± 8.7	7.7 (4.2–11.3)	0.003
Engineering Ethics (b14)	76.8 ± 10.5	84.1 ± 7.9	7.3 (3.8–10.8)	0.004
Industry Case Updates (b7)	82.3 ± 9.4	88.6 ± 6.2	6.3 (2.9–9.7)	0.012

## Data Availability

The data presented in this study are available on request from the corresponding author. The data are not publicly available due to privacy concerns for the participants involved in the study.

## Abbreviations

The following abbreviations are used in this manuscript:

MCDM	Multi-Criteria Decision-Making
AHP	Analytic Hierarchy Process
IVIF-AHP	Interval-Valued Intuitionistic Fuzzy Analytic Hierarchy Process
IVIFN	Interval-Valued Intuitionistic Fuzzy Number
FCE	Fuzzy Comprehensive Evaluation
KNN	K-Nearest Neighbors
WAA	Weighted Arithmetic Averaging
BEI	Building Electrical and Intelligent Engineering
BPSDT	Building Power Supply and Distribution Technology
